# Techno‐economic analysis of a solar‐assisted heat pump dryer for drying agricultural products

**DOI:** 10.1002/fsn3.3810

**Published:** 2023-11-09

**Authors:** Evordius Laurent Rulazi, Janeth Marwa, Baraka Kichonge, Thomas T. Kivevele

**Affiliations:** ^1^ School of Materials, Energy, Water and Environmental Sciences (MEWES) Nelson Mandela African Institution of Science and Technology (NM‐AIST) Arusha Tanzania; ^2^ Tanzania Commission for Science and Technology (COSTECH) Dar es Salaam Tanzania; ^3^ School of Business Studies and Humanities (BuSH) Nelson Mandela African Institution of Science and Technology (NM‐AIST) Arusha Tanzania; ^4^ Department of Mechanical Engineering Arusha Technical College (ATC) Arusha Tanzania

**Keywords:** fruits and vegetables, solar‐assisted heat pump dryer, techno‐economic analysis

## Abstract

Postharvest losses (PHLs) of biomaterials, such as vegetables and fruits, significantly impact food security and economic stability in developing nations. In Tanzania, PHLs are estimated to range between 30% and 40% for cereal crops and even higher for perishable crops such as fruits and vegetables. Open‐sun drying (OSD) is the most extensively employed method because of its affordability and simplicity. However, OSD has several drawbacks, including difficulties in managing drying parameters, long drying times owing to adverse weather, and product contamination. The solar‐assisted heat pump dryer (SAHPD) is a technology designed as an alternative solution for drying biomaterials and reducing PHL. A limited number of SAHPDs have been constructed in developing nations. Most of the works have concentrated on the performance analysis of the systems. This neglects the techno‐economic assessment, which is important to provide both a quantitative and qualitative understanding of the financial viability of the technology. The present study therefore investigates the techno‐economic analysis of a novel SAHPD for drying agricultural products, particularly vegetables and fruits. To determine whether the SAHPD technology is technically and economically viable, tomatoes and carrots were dried and analyzed to determine their thermal and economic performance. The results show that the initial moisture contents of tomatoes (*Lycopersicum esculentum*) and carrots (*Daucus carota*) were reduced from 93% and 88% to 10% in 11 and 12 h, respectively. The coefficient of performance (COP), drying time (DT), specific moisture extraction ration (SMER) and thermal efficiency ($\eta_{T}$) were found to be 3.4, 2.3 kg/h, 1.33 kg/kWh and 54.0%, respectively. The economic analysis was assessed using the annualized cost, lifecycle savings, and payback period for the dryer's life span of 15 years. The initial investment of the SAHPD was $5221.8 and the annualized cost was $1076.5. The cumulative present worth for 15 years was found to be $23,828.8 and $27,553.1 for tomatoes and carrots, respectively. The payback period for tomatoes was found to be 3 years, whereas for carrots it was 2.6 years. Based on thermal and economic performance assessment results, the developed SAHPD is technically and economically viable to be considered for further investments.

## Graphical abstract

Solar‐assisted heat pump drying technology is a drying technology designed for the drying of agricultural products, hence reducing postharvest losses, especially in developing countries. This research article therefore investigates the performance as well as the economic analysis of the designed solar dryer for drying vegetables and fruits in Tanzanian environment.

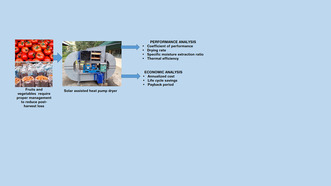

**Nomenclature**
$C_{a}$
: Annualized capital cost (USD)
$C_{b}$
: Selling price of 1 kg of dried product (USD)
$C_{\mathrm{ds}}$
: Total cost of drying 1 kg of product (USD)
$C_{m}$
: Annualized maintenance cost (USD)
$C_{\mathrm{rf}}$
: Running fuel cost (USD)
$C_{\mathrm{ds}}$
: Total cost of drying 1 kg of product (USD)
$C_{\mathrm{re}}$
: Cost of electricity (USD)
$C_{s}$
: Cost of drying 1 kg of dried product (USD)
$C_{\mathrm{fp}}$
: Cost of fresh product (USD/kg)
DR: Drying Rate (kg/h)
DT: Drying Time (h)
*d*
: Interest rate (%)
*D*
: Number of drying days in a year (days)
$D_{d}$
: Number of days used to dry one batch of the product (days)
$F_{c}$
: Capital recovery factor
$F_{\mathrm{pi}}$
: Present worth factor
h
: Specific enthalpy (kJ/kg)
*i*
: Inflation rate (%)
*n*
: Number of years
$M_{y}$
: Weight of product dried in the dryer (kg)
$M_{f}$
: Weight of fresh product (kg)
$M_{w}$
: Moisture loss from the drying product (kg)
PP: Payback period (years)
$P_{j}$
: Present worth of savings (USD)
${\dot{Q}}_{\text{Cond}}$
: Heat delivered in condenser
RH: Relative humidity (%)
$S_{\mathrm{kg}}$
: Savings per kilogram of the dried product (USD)
$S_{j}$
: Year‐wise savings of the dried product (USD)
$S_{1}$
: Savings during first year
$t_{d}$
: Drying time (h)
$V_{s}$
: Annualized Salvage value (USD)
${\dot{W}}_{\text{Comp}}$
: Power input to the compressor (kW)
$W_{\mathrm{fan}}$
: Fan input power (W)

## 1 INTRODUCTION

Agriculture, an important economic sector in developing Sub‐Saharan African countries, suffers from food crop postharvest losses (PHLs). PHLs of fruits and vegetables in Sub‐Saharan Africa range from 30% to 80%, depending on the type of crop and location (James & Zikankuba, 2017). In Tanzania, the PHLs of food cereal crops were estimated to range between 20% and 40% in 2019 (Maziku, 2019) and more than 40% for vegetables and fruits (Dome & Prusty, 2017). According to the Ministry of Agriculture (2019), PHLs are caused by different factors such as pest infestation, poor transportation infrastructures, improper storage practices, improper harvesting and drying practices, improper weighing and packaging, a lack of reliable markets, a lack of appropriate processing technologies, and a lack of farmer's knowledge on postharvest management along the value chain. Fruits and vegetables are important agricultural products in our bodies because they provide essential nutrients such as proteins, vitamins, carbohydrates, and minerals and also significantly boost monetary exchange in local communities. However, their high perishability makes fruits and vegetables vulnerable to PHLs. The situation necessitates the development and implementation of effective yet low‐cost technologies to significantly reduce food crop PHLs. According to Gunathilake et al. (2018), there are three common methods available for food preservation, namely, canning, freezing, and drying. Drying is considered the best method because it preserves agricultural products, extends shelf life, reduces volume and weight, and facilitates easy transportation, storage, and packaging of the products (Calín‐Sánchez et al., 2020). Drying is one of the mitigation measures being used to reduce PHLs by lowering crop moisture content to a safe storage level.

Open‐sun drying (OSD) is one of the oldest and most widely used traditional methods due to its ease of use and low cost (Sontakke & Salve, 2015; Vaghela et al., 2018). However, OSD has a number of drawbacks, such as difficulties in controlling drying kinetics (Pochont et al., 2020), long drying times (Natarajan et al., 2019), product contamination by insects, birds, and bad weather (Singh et al., 2019), loss of natural aesthetics and minerals, as well as large drying area and labor (Zachariah et al., 2021). Solar drying technologies can provide a cost‐effective and efficient way to preserve food as well as reduce PHLs, particularly in developing countries.

Given current trends toward scarce and expensive conventional energy and uncertainty about their future, the combination of heat pump dryers and solar energy is becoming the most economically viable option for drying agricultural products. Solar‐assisted heat pump dryers (SAHPDs) are preferred over other type of dryers due to their economical, reliable and good quality of the dried products (Şevik, 2014). The SAHPD is one of the technologies designed to overcome the limitations of passive solar dryers, which depend entirely on solar radiation, and other active drying technologies, which consume sufficient energy (Salehi, 2021). SAHPD can function successfully throughout the day, even when there is no sunlight, assuring the quality of the dried products (Gan et al., 2017). Loemba et al. (2022) conducted a comprehensive assessment of heat pumps for drying agricultural products and reported that using heat pumps significantly reduced drying time, improved specific moisture extraction, and reduced energy consumption.

The great bulk of research on the utilization of SAHPD for drying agricultural products has been on performance metrics. For example, Singh et al. (2020a) conducted a performance comparison analysis for the SAHPD and heat pump dryer (HPD) for drying banana chips. They reported that SAHPD performed better in terms of energy efficiency and specific moisture extraction ratio (SMER) compared to HPD alone due to the combination of both HPD and solar energy. In comparison with other solar drying technologies, SAHPD has been reported to reduce drying time and improve efficiency. Yahya et al. (2016) conducted a performance evaluation of the solar dryer (SD) and SAHPD for drying cassava and reported that the drying time using the SAHPD was 9 h, whereas the SD was 13 h. It was also found that the thermal efficiency and SMER for SAHPD were 30.9% and 0.47 kg/kWh, respectively, whereas for SD, the thermal efficiency and SMER were 25.6% and 0.38 kg/kWh, respectively. Yahya et al. (2018) conducted performance and economic analysis for the SAHPD fluidized bed integrated with biomass for rice drying and obtained an average SMER of 0.24 kg/kWh, with thermal dryer efficiency ranging 8.4%–25.6%. SAHPD has been reported to have a high coefficient of performance (COP), which is associated with lower operation costs for the dryer. Qiu et al. (2016) conducted a performance analysis of the SAHPD for the drying of radish, pepper, and mushrooms, and it was reported that the COP varied from 3.21 to 3.49.

Most of the studies on heat pumps have focused on the improvement of the performance of SAHPD and HPD, with few studies addressing the aspect of economic analysis on payback periods. Economic analysis is a very important aspect of the solar dryer because it provides necessary information on whether the proposed dryer is economically feasible, cost‐effective, sustainable, and likely to be adopted and considered for further investment. Some of the few studies presented on economic analyses of SAHPD for drying agricultural products. Yahya et al. (2018) estimated a short payback period of 1.6 years for a solar‐assisted heat pump fluidized bed dryer coupled with a biomass furnace, specifically designed for rice drying. In another study, Singh et al. (2020a) reported a payback period of 3.8 years for a SAHPD designed to dry banana chips. Qiu et al. (2016) conducted a performance and economic analysis of the heat recovery and thermal storage of a SAHPD for drying radish, pepper, and mushrooms. Their findings indicated that the cumulative net present value for radish was $10,182, whereas for pepper it was $16,072, and mushroom it was $29,749. It was also reported that the payback periods were 6, 4, and 2 years for radish, pepper, and mushroom, respectively. These various studies shed light on the economic feasibility of SAHPD in different agricultural contexts.

To the authors’ knowledge, a study has yet to be conducted on the techno‐economic assessment of SAHPD for drying tomatoes and carrots in the African context. Climatic conditions play a vital role in the performance of solar‐assisted heat dryers. Therefore, this study aims to investigate the performance and techno‐economic feasibility of a novel SAHPD for drying vegetables and fruits, specifically tomatoes and carrots, in the context of Tanzania. Tomatoes, which are botanically classified as fruits, and carrots, which are root vegetables, are essential products in Tanzania's economy as well as a source of nutrition for our health. However, they are vulnerable to post‐harvest loss, especially during peak seasons.

## 2 MATERIALS AND METHODS

### 2.1 Materials and equipments

The SAHPD consist of heat pump components such as a condenser, evaporator, compressor, and expansion valves which are integrated with a fabricated component such as a solar collector, air duct, drying chamber, and support stand to form a complete dryer. The materials used for the fabrication of the solar dryer were bought from the local market. Some of the materials used are mild steel sheet of 1.5 mm thick, mild steel angle iron of 30 × 30 × 3 mm, clear transparent glass of 6 mm thickness, insulation materials (glass wool), and bolts and nuts.

#### 2.1.1 The heat pump components

The heat pump components were procured from the local market and subsequently integrated with other custom‐made components. Table 1 shows the specifications of heat pump components.

**TABLE 1:** Specifications of heat pump components.

S/n	Name of the component	Specifications
1	Danfoss reciprocating compressor	4 HP (MTZ36GJ5EVE, LR70, low‐pressure side 22.6 bar, high pressure side 29.4 bar)
2	Fan Power	175 W, 0.75 A, and 1335 rpm
3	Condenser	4 HP with a size of 1020 × 450 × 300 mm
4	Evaporator	4 HP with a size of 1020 × 510 × 400 mm
5	Expansion valve	R134a Danfoss (TEN2 R134a,068Z3348, −40/+10°C/−40/+50°F)

#### 2.1.2 Drying chamber

The drying chamber was constructed using 1.5 mm thick mild steel sheets and reinforced with 25 × 25 × 3 mm angle iron for added strength. At the top of the drying chamber, a solar collector was seamlessly integrated. To minimize heat exchange between the inside and outside of the chamber, a 25 ‐mm‐thick layer of glass wool insulation was applied. Inside the drying chamber, a black paint coating was applied to efficiently absorb solar heat energy. The design of the drying chamber allowed for the drying of 30 kg of vegetables or fruits per batch.

#### 2.1.3 Solar collector

The solar collector was constructed using 1.5 ‐mm‐thick mild steel. On top of it, a solar collector panel measuring 946 × 946 mm was installed to capture solar radiation. The tilt angle of the solar collector was carefully designed to ensure it received an adequate amount of solar radiation, as determined by the study conducted by Missana and Mashingo (2022). Their findings indicated an optimal tilt angle of 13.4° for efficient solar radiation capture.

#### 2.1.4 Air duct

A flexible air duct was employed to facilitate the flow of air between the evaporator, condenser, and drying chamber. This duct was crafted from 1.5 mm thick mild steel and strengthened with 25 × 25 × 3 mm angle iron. The air duct possessed an elliptical shape with a square cross section measuring 480 × 480 mm. To minimize heat loss, it was additionally insulated with a 25 ‐mm layer of fiberglass. Figure 2 provides a visual representation of some of the key features of the solar‐assisted heat pump dryer.

### 2.2 Experimental setup

**FIGURE 1:**
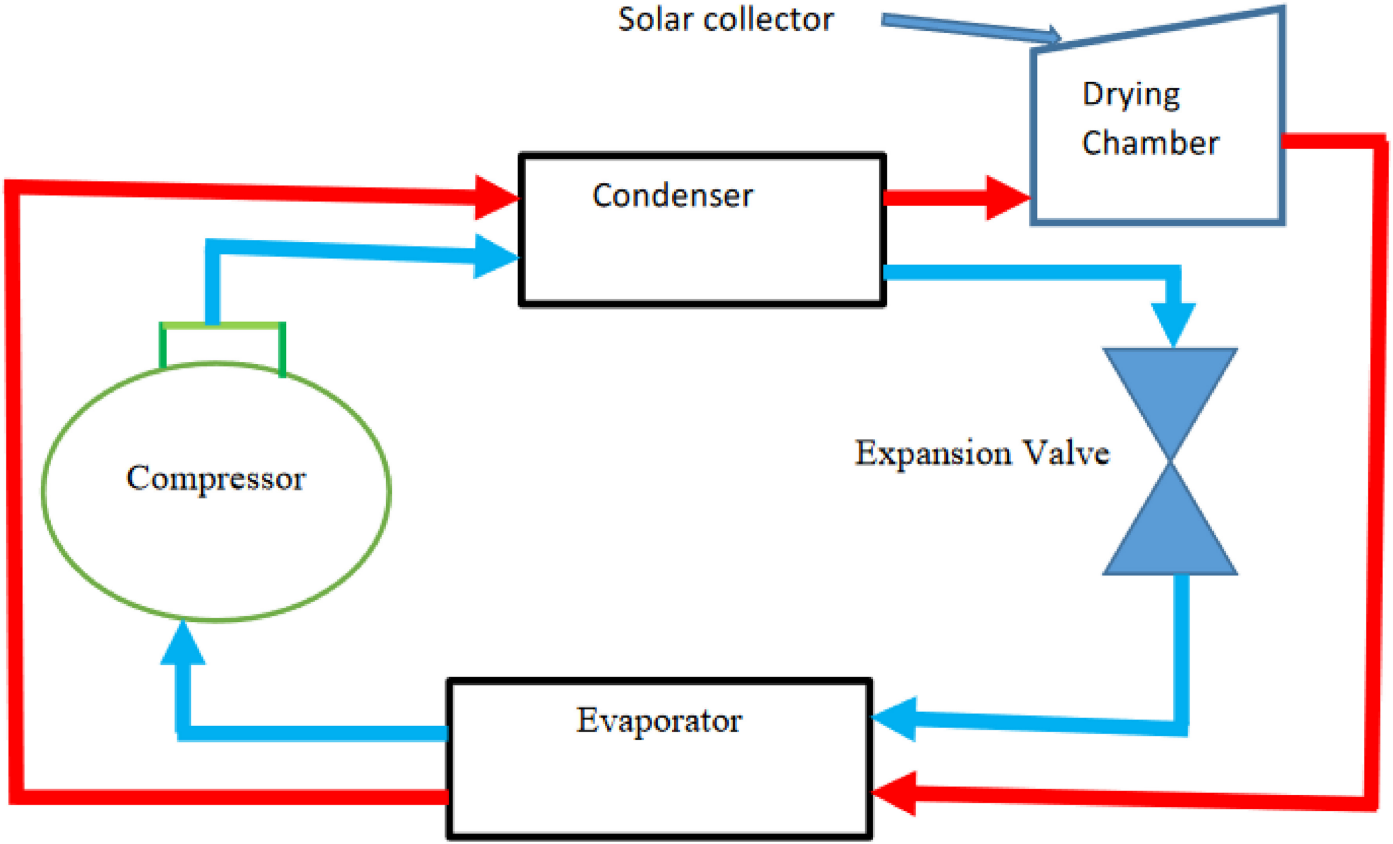
Schematic diagram of a solar‐assisted heat pump drying system.

**FIGURE 2:**
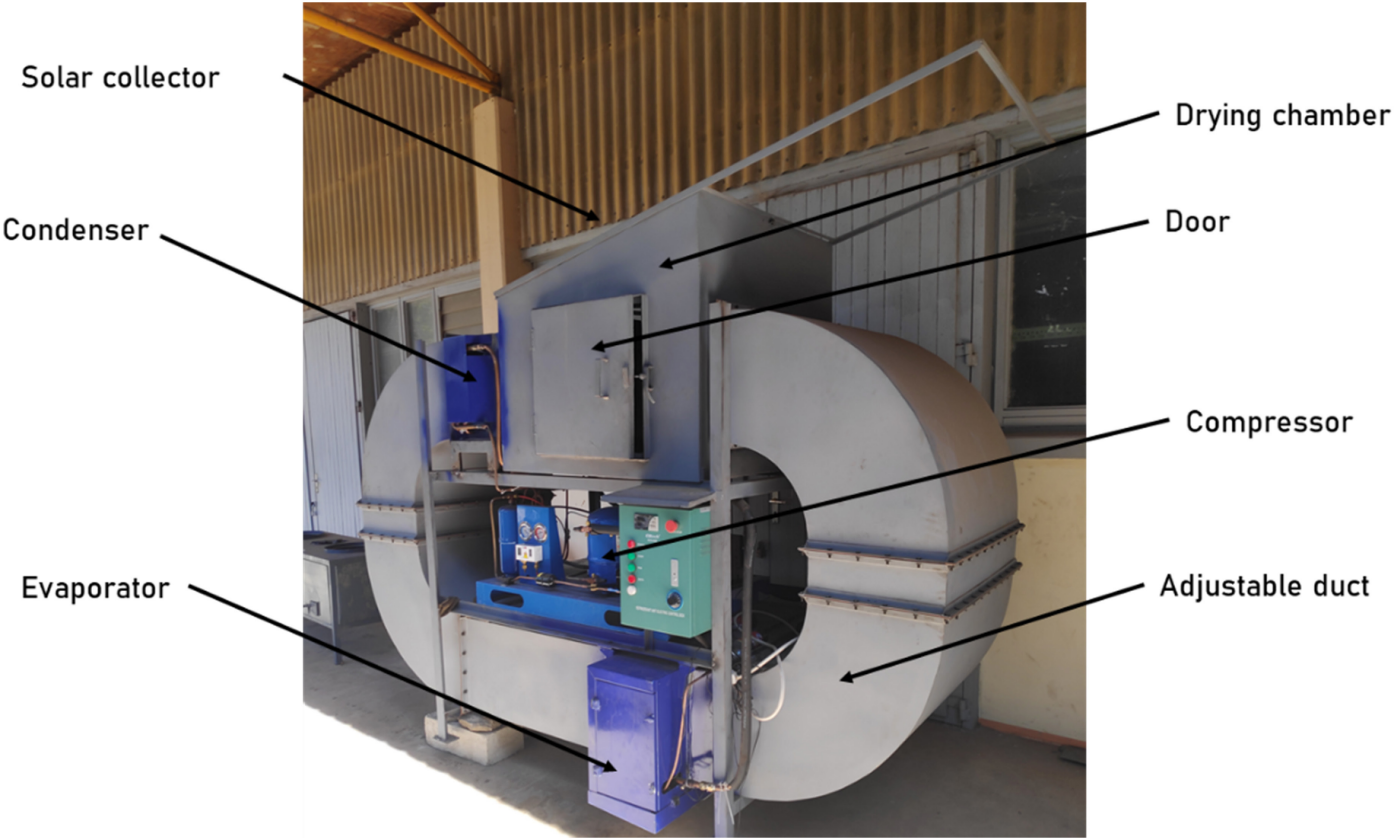
Photograph of the developed experimental setup of a solar‐assisted heat pump dryer.

A SAHPD was designed, fabricated, and tested at the Workshop of Mechanical Engineering Department, Arusha Technical College (ATC), located in Arusha Municipality, Tanzania. The compressor used in this study was a single‐phase compressor manufactured by Danfoss Company, and the working fluid was R‐134a. The specification of the heat pump system is shown in Table 1. Figures 1 and 2 show the schematic and photography of the SAHPD, respectively. In Figure 1, the red line shows the route of drying air in the air duct, while the blue line shows the route of the refrigerant. There are four main components in this heat pump solar drying system: a compressor, a condenser, an evaporator, and an expansion valve.

### 2.3 Experimental procedure

The experiments were carried out on tomatoes (*Lycopersiconesculentum*) and carrots (*Daucus carota*). The samples were purchased at a local market in Arusha Region, Tanzania, washed, and sliced into about 4 mm‐thick pieces. According to Krishnan and Sivaraman (2017), a slice thickness of 3–4 mm is considered the optimum size for drying. A sample of 30 kg of tomatoes and 30 kg of carrots were dried at a different time on both SAHPD and open sun. The data were recorded every 30 min until the mass change of the sample became insignificant.

Three replicates were conducted for each experiment, and the average value was calculated. Several parameters were monitored throughout the experiments to assess the dryer's performance. These measurements and data collection methods were essential for evaluating the performance and efficiency of the solar‐assisted heat pump dryer during the experiments.

- *Solar Irradiance*: Solar irradiance was measured using a TES 132 Solar power meter. A sensor probe was placed on the surface of the solar collector to capture this data.
- *Weight of Drying Products*: The weight of the drying products was determined using the FF1976 Constant digital weighing scale, which was designed with a carrier on top for holding the drying products.
- *Pressure*: Pressure on the high and low‐pressure sides was measured using XMK pressure gauges.
- *Temperature and Humidity*: Temperature and humidity inside the drying chamber were monitored using an SSN‐22E USB temperature and humidity data logger meter. Three sensor probes were inserted into different positions within the drying chamber to record this data. Additionally, two similar sensor probes were located outside to measure temperature and humidity in the surrounding environment.
- *Compressor Parameters*: Voltage and current values of the compressor were recorded using an Agilent U1212A Clamp meter. These values were used to calculate the power consumed by the compressor and the overall power consumption of the heat pump system.

### 2.4 Measuring instruments, error, and uncertainty analysis

The instruments used for measurements during experiments, the range of their measurements, accuracy, and resolution are shown in Table 2. Regardless of how precise and accurate a measurement is, it is typically subject to some degree of uncertainty. The two main contributors to these uncertainties are measurement techniques, sometimes known as random errors and measuring apparatus, commonly known as systematic errors. The total errors in measurement were calculated by using Equation (1) according to Gulcimen et al. (2016).

(1) $$\;w_{\mathrm{th}}=\sqrt{{\left(W_{1}\right)}^{2}+{\left(W_{2}\right)}^{2}\ldots ..\ldots {\left(W_{\infty }\right)}^{2}}$$

where; W = independent variable affecting measurement.

**TABLE 2:** Measuring instruments and uncertainties in measurements.

S/n	Instrument	Range	Accuracy	Resolution	Error (%)	Uses
1	SSN‐11E USB temperature data logger meter	−40–125°C	±0.5°C	±0.1°C	0.1414	Temperature measurement
2	SSN‐22E USB temperature/humidity data logger meter	0–100% RH	±0.3 RH	±0.1 RH	0.1414	Humidity measurement
		−40–125°C	±0.3°C	±0.1°C	0.1414	Temperature measurement
3	TES 132 Solar power meter	0–2000 W/m^2^	±10 W/m^2^	0.1 W/m^2^, 1 W/m^2^	0.1414	Solar radiation measurement
4	Kestrel 3000 wind meter	0.6–40 m/s	±0.1 m/s	0.05 m/s	0.0707	Wind measurement
5	FF1976 Constant Digital weighing scale	0–40 kg	±0.14 g	0.1 g	0.1414	Weight measurement
6	Agilent U1212A Clamp meter	0–600 V	± (1.5% +3)	0.1 V	0.1414	Voltage measurement
		0–400 A	± (2.5% +3)	0.1 A	0.1414	Current measurement
7	XMK Antihunting oil gauge	1–18 MPa	±0.1	0.1 psi	0.1414	Pressure measurements

The independent variables affecting measurements were determined by using Equation (2) according to AR and Veeramanipriya (2022).

(2) $$w_{n}=\sqrt{{\left(W_{\text{instument}}\right)}^{2}+{\left(W_{\text{reading}}\right)}^{2}}$$

The overall errors in measurement of different parameters were given by Equation (3)

(3) $$w_{\text{Total}}=\sqrt{{\left(W_{\text{temperature}}\right)}^{2}+\left(W_{\text{humidity}}\right)+\left(W_{\text{solar}}\right)+\left(W_{\text{wind}}\right)+\left(W_{\text{scale}}\right)+{\left(W_{\text{pressure}}\right)}^{2}}$$

The combined uncertainties in the measuring devices and reading errors were determined using Equation (3) and were found to be approximately ±1.3% (rounded). This value is quite small when compared to the acceptable range of ±10% as established by Choi et al. (2018).

### 2.5 SAHPD performance

SAHPD performance was evaluated by determining the moisture content (*M*_c_), weight of water evaporated from the product (*M*_w_), drying rate (DR), COP, and SMER.

The moisture content was determined by using the gravimetric oven drying method, in which 10 g of paste was accurately weighed in a clean, dry petridish and the weight of the sample was recorded. The petridish was then dried in an oven at 105°C for 24 h until a constant weight was obtained. Then, the petridish was placed in the desiccator for 30 min for cooling and then reweighed again. The percentage of moisture content was calculated using Equation 4.

(4) $$M_{c}\;\left(\mathrm{On}\;\mathrm{wet}\;\text{basis}\right)=\frac{\text{Total moisture content}}{\text{Total weight of product}}$$

The weight of water evaporated from the drying products (*M*_w_) was calculated using Equation (5) as proposed by Fudholi et al. (2015).

(5) $$M_{w}=\frac{M_{o}\left(M_{i-}M_{f}\right)}{100-M_{f}}$$

where $M_{o}$ = Initial mass of the products; $M_{i}$ = Initial moisture content of the product on the wet basis; $M_{f}$= Final moisture content of the product on wet basis.

The drying rate for carrot and tomato was calculated using Equation (6) as given by Hasibuan et al. (2020)

(6) $$\mathrm{DR}=\frac{M_{w}}{t}$$

where $M_{w}$ = Mass of water evaporated from the drying products (kg); t = Drying time (h).

SMER describes the ratio of the moisture removed (*M*_w_) from the drying product over the total energy input to the system. It was calculated using Equation (7) as proposed by Hasibuan et al. (2020))

(7) $$\text{SMER}=\frac{M_{w}}{\left(I_{T}A_{\mathrm{sc}}+W_{\text{comp}}+W_{b}\right)t}$$

where $M_{w}$ is the weight of water evaporated from the product (kg); $I_{T}$ = Solar radiation incident to the collector; $A_{\mathrm{sc}}$ = Collector area (m^2^); $W_{\text{comp}}$ = Electrical energy consumed by the compressor; $W_{b}$ = Electrical energy consumed by the fans.

The COP is the ratio of usable heat or heat energy released by the refrigerant in the condenser to the electrical energy required by the compressor. It was calculated using Equation (8) as proposed by (Singh et al., 2020b).

(8) $${\mathrm{COP}}_{\mathrm{HP}}=\frac{h_{2}-h_{3}}{h_{2}-h_{1}}$$

$h_{2}$= Enthalpy (kJ/kg) of the superheated vapor from the compressor; $h_{1}$ = Enthalpy (kJ/kg) of the saturated vapor state from the evaporator; $h_{3}$ = Enthalpy (kJ/kg) of the saturated liquid state from the condenser.

The pressure (kPa) and temperature (°C) for refrigerant for sanction (low) and sanction side (high) was obtained from the pressure gauges, whereas enthalpies in (kJ/kg) were obtained from the refrigerant R134a thermodynamic table and pressure‐enthalpy chart.

**FIGURE 3:**
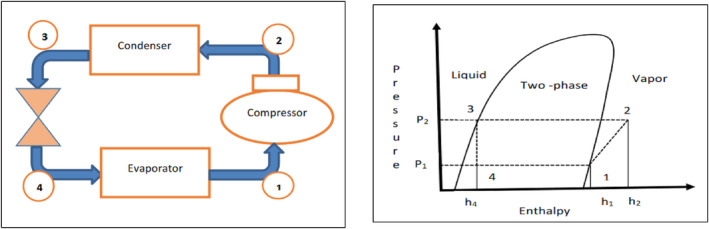
Schematic and pressure‐enthalpy diagram.

Figure 3 illustrates the path of the refrigerant and its various states as it undergoes transitions between liquid, liquid‐to‐vapor, and vapor phases within the system. At the initial point of entry, denoted as “Point 1,” into the compressor, the refrigerant is observed to be in a supersaturated vapor condition. Within the compressor, its pressure undergoes a significant increase, evident in the pressure‐enthalpy diagram as it moves from “P1” to “P2.” As it exits the compressor at “Point 2,” the refrigerant is in a superheated vapor state. Subsequently, the refrigerant proceeds from “Point 2” to “Point 3,” where it releases heat into the drying chamber and leaves the condenser in a fully saturated liquid state, completing this phase of the cycle. The high‐pressure liquid then enters the expansion chamber, where it experiences a pressure drop from “P2” to “P1” due to the expansion process. Upon exiting the expansion valve at “Point 4,” the refrigerant exists in a two‐phase state, comprising a mixture of liquid and vapor. It then enters the evaporator, where it absorbs heat from the surrounding environment. During this stage, all the liquid transforms into a supersaturated vapor state, continuing the cycle. This intricate process of phase changes and heat exchange within the system is integral to facilitating the drying process efficiently.

The thermal efficiency of the drying system was determined by using Equation 9, according to Hasibuan et al. (2020).

(9) $$\eta_{T}=\frac{M_{w}H_{\mathrm{fg}}}{I_{T}A_{\mathrm{sc}}+W_{\text{comp}}+W_{f}}$$

where $M_{w}$ = Weight of water evaporated from product (kg); $I_{T}$ = Solar radiation incident to the collector (W/m^2^); $A_{\mathrm{sc}}$ = Collector area (m^2^); $H_{\mathrm{fg}}$ = Latent heat of vaporization was obtained from a table of saturation properties for steam temperature at an average drying temperature; $W_{\text{comp}}$ = Electrical energy consumed by the compressor; $W_{f}$= Electrical energy consumed by fan.

### 2.6 SAHPD economic analysis

The economic analysis of the SAHPD was evaluated using three economic standard methods, namely, annualized cost, life cycle savings, and payback period. Some assumptions about the parameters were made for the evaluation of the economic analysis of the dryer; some were based on the Tanzanian economic scenario, and others on previous studies. These includes the fact that the life span of a solar dryer was assumed to be 15 years. Inflation rates of 4.8% were adopted from the monthly economic review report from the Bank of Tanzania (2023). Further, the 7% annual interest rate stated by Aly et al. (2019) was adopted. Since the SAHPD requires minimal service, the maintenance cost and salvage value were considered to be 5% of the annualized capital cost, as utilized in the study by Philip et al. (2022). Table 3 shows a summary of the important parameters used during the analysis.

**TABLE 3:** Summary of parameters for economic analysis of the solar‐assisted heat pump dryer (SAHPD).

S/n	Parameters	Value
1	Capital investment cost	$5221.8
2	Maintenance cost	5% of annual capital cost
3	Salvage value	5% of annual capital cost
4	Inflation rate (*i*)	4.8%
5	Dryer life span (*n*)	15 years
6	Cost of fresh tomatoes	$0.15/kg
7	Selling price of the dried tomatoes	$4/kg
8	Cost of fresh carrots	$0.2/kg
9	Selling price of the dried carrots	$3/kg
10	Interest rate $\left(\;d\right)$	7%
11	Number of days of operation per year (D)	300 days
12	Dryer drying capacity	30 kg/batch/day
13	Cost for electricity	0.155 $/kWh

#### 2.6.1 Annualized cost

The annualized cost method estimates the cost of drying products on an annual basis. The annualized cost was calculated according to Philip et al. (2022) by using Equation 10.

(10) $$C_{a}=C_{\mathrm{ac}}+C_{m}-V_{s}+C_{\mathrm{rf}}+C_{\mathrm{re}}$$

where $C_{a}$ = Annualized cost of the dryer (USD); $C_{\mathrm{ac}}$= Annualized capital cost (USD); $C_{m}$ = Annualized maintenance cost (USD); $V_{s}$= Annualized salvage value (USD); $C_{\mathrm{rf}}$= Running fuel cost (USD); $C_{\mathrm{re}}$ = Cost of electricity (USD).

(11) $$C_{\mathrm{ac}}=C_{\mathrm{cc}}F_{c}$$

where $C_{\mathrm{cc}}$= Capital investment cost of the dryer (USD); $F_{c}$= Capital recovery factor.

(12) $$V_{s}=VF_{s}$$

where *V* = Salvage value (USD); $F_{s}$ = Salvage fund factor.

(13) $$F_{c}={\frac{d\;\left(1+d\right)}{{\left(1+d\right)}^{n}-1}}^{n}$$

(14) $$\;F_{s}=\frac{d}{{\left(1+d\right)}^{n}-1}$$

where d= Interest rate; n = Lifespan of solar dryer.

Weight (kg) of the dried product in the dryer is given by Equation 15 according to Philip et al. (2022).

(15) $$M_{y}=\frac{DM_{d}}{D_{b}}$$

where D = Number of drying days in a year; $M_{d}$= Weight (kg) of dried product removed from the dryer per batch; $\;D_{b}$= Number of days for drying one batch of the product.

The cost for drying 1 kg of the dried products ($\;C_{s}$) is given by Equation 16 according to Philip et al. (2022).

(16) $$\;C_{s}=\frac{C_{a}}{M_{y}}$$

The running fuel cost $\left(C_{\mathrm{rf}}\right)$ for this particular dryer is assumed to be zero because the source of energy to power the compressor of the heat pump is electricity. The running cost of electricity was calculated using Equation (17) according to Philip et al. (2022).

(17) $$\;C_{\mathrm{re}}=R\times W\times C_{e}$$

where $C_{e}$= Unit charge of electricity; R = Number of hours of running a heat pump in a year; W = Power supplied to the system.

#### 2.6.2 The life cycle savings

The method describes how much can be saved on a drying basis and then projects the present value of annual savings over the dryer's lifespan (Philip et al., 2022). The cost of fresh product per kilogram of dried product was calculated using Equation 18, according to Chavan and Thorat (2021).

(18) $$\;C_{\mathrm{dp}}=C_{\mathrm{fp}}\times \frac{\;M_{f}}{\;M_{d}}$$

where $C_{\mathrm{fp}}$ = Cost of fresh product (USD/kg); $M_{f}$= Weight of fresh product (kg).

The total cost of drying 1 kg of the product $\;\left(C_{\mathrm{ds}}\right)$is given by the sum of the total cost of fresh product ($\;C_{\mathrm{dp}}$), and the cost required to dry 1 kg of the product ($\;C_{s}$) was calculated using Equation 19 according to Chavan and Thorat (2021).

(19) $$C_{\mathrm{ds}}=C_{\mathrm{dp}}+C_{s}$$

Considering the dried products by dryer for this particular project are to be branded, the savings per kilogram of the dried product ($S_{\mathrm{kg}}$)in annual basis are expressed in Equation 20, according to Prakash et al. (2017).

(20) $$\;S_{\mathrm{kg}}=C_{b}-C_{\mathrm{ds}}$$

where $\;C_{\mathrm{ds}}$ = Total cost of fresh product; $C_{b}$ = Selling price of 1 kg of dried product (USD).

Batch‐wise savings ($S_{b}$) on drying days are expressed in Equation 21, according to Prakash et al. (2017).

(21) $$S_{b}=S_{\mathrm{kg}}\times M_{d}$$

$S_{\mathrm{kg}}$ = The savings per kilogram of dried products on an annual basis.

Daily‐wise savings $\left(S_{d}\right)$ on drying days is expressed in Equation 22, according to Prakash et al. (2017).

(22) $$S_{d}=\frac{S_{b}}{D_{b}}$$

$D_{b}$ = Number of days for drying one batch of the product.

#### 2.6.3 Annual saving's present worth

Year‐wise savings of the drying products $\left(S_{j}\right)$ under study in the *n*th year are expressed in Equation 23 as proposed in Prakash et al. (2017).

(23) $$S_{n}=S_{d}\times D\times {\left(1+i\right)}^{n-1}$$

where *I* = Inflation rate (%); $\;F_{\mathrm{pn}}\times S_{n}D$ = Number of drying days in a year; $S_{d}$ = Savings per day (USD).

The present worth of savings in the *n*th year is given by Equation 24, according to Prakash et al. (2017).

(24) $$\;P_{n}=F_{\mathrm{pn}}\times S_{n}$$

where $F_{\mathrm{pn}}$ = Present worth factor for the *n*th year (USD); $S_{n}$ = Yearly savings for drying product in *n*th year (USD).

The present worth factor for the *n*th year is given by Equation 25, according to Prakash et al. (2017).

(25) $$\;F_{\mathrm{pn}}=\frac{d}{{\left(1+d\right)}^{n}}$$

where d = Interest rate and *n* = number of years.

Life cycle savings is found by summation of yearly savings of present worth over life span of the dryer.

#### 2.6.4 Payback period (PP)

The payback period (PP) was calculated by using Equation (26) according to Prakash et al. (2017).

(26) $$\mathrm{PP}=\frac{\ln\;\left(1-\frac{C_{\mathrm{cc}}\;}{S_{1}}\left(d-i\right)\right)}{\ln\;\left(\frac{1+i}{1+d}\right)}$$

where d = Annual interest rate = 7%; *i* = Inflation rate = 4.8%; $S_{1}$ = Savings during the first year; $C_{\mathrm{cc}}$ = Capital investment cost of the dryer (USD).

## 3 RESULTS AND DISCUSSION

### 3.1 Performance evaluation

**FIGURE 4:**
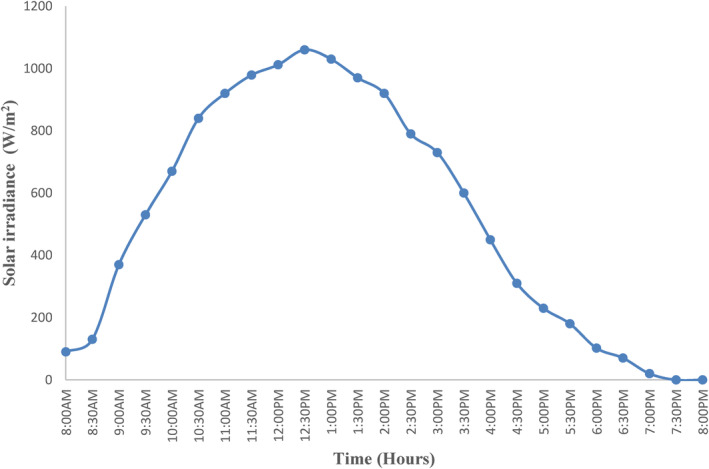
Variation of solar radiation with time.

The experiments were carried out between May and November 2022, from 8:00 a.m. to 8:00 p.m. This is the period when there is a sufficient amount of sun radiation in the Arusha Region. The solar radiation ranged from 90 W/m^2^ to 1060, and the minimum irradiation was observed during the morning and evening, whereas the maximum irradiation was observed around noon. Figure 4 depicts the time‐dependent fluctuations in mean hourly solar irradiance (W/m^2^) during drying experiments. The minimum irradiations were observed during the morning and evening, whereas the maximum irradiations were observed around 12.30 p.m.

**FIGURE 5:**
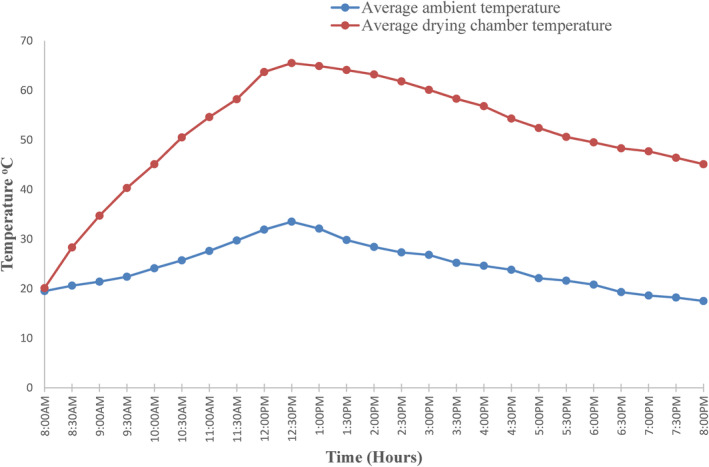
Variation of ambient and temperature in the drying chamber.

Figure 5 shows the relationship between the average temperature inside the drying chamber, ambient temperature, and solar radiation. The startup of the system was done at 8:00 a.m., and the heat pump was set to a temperature of 50°C and an average air velocity of 1.5 meters per second because drying products using these parameters maintains vital components such as protein, vitamins, and minerals, as reported by Das Purkayastha et al. (2013) for tomatoes and Gojiya and Vyas (2015) for Kothimda product. In addition, a temperature range of 45–60°C is safe for drying heat‐sensitive products such as fruits and vegetables and ensuring the quality of the dried products (Sontakke & Salve, 2015). The temperature inside the drying chamber gradually started to increase until it reached a maximum average of 65°C around 12:30 p.m. during the time of peak solar irradiance. The sliding gate with fiber wood that was installed on the top of the solar collector was designed to restrict the development of excess temperature in the drying chamber and was closed when the temperature rose above 65°C. When the solar radiation was not available, the maximum temperature attained by the heat pump alone was 51°C. In this case, the heat energy from the solar collector was contributing to a rise in temperature of about 14°C, which is about 21.5% of the total maximum temperature recorded in the drying chamber. However, the temperature inside the drying chamber was in the range of 45–65°C, depending on the intensity of solar radiation. This result is in agreement with Yahya (2016), who used SAHPD integrated with a biomass furnace for drying red chilli. In that study, it was found that the solar collector's contribution to heat energy ranged from 2.7% to 30%, while the condenser's contribution ranged from roughly 43.7% to 50.4%. The current study also agrees with Yahya et al. (2016), who contrasted solar dryer and SAHPD for drying cassava and reported that the average contribution of energy from solar collector was 44.6%. Based on these observations, solar collector plays important role of supplying additional energy to the system for drying.

**FIGURE 6:**
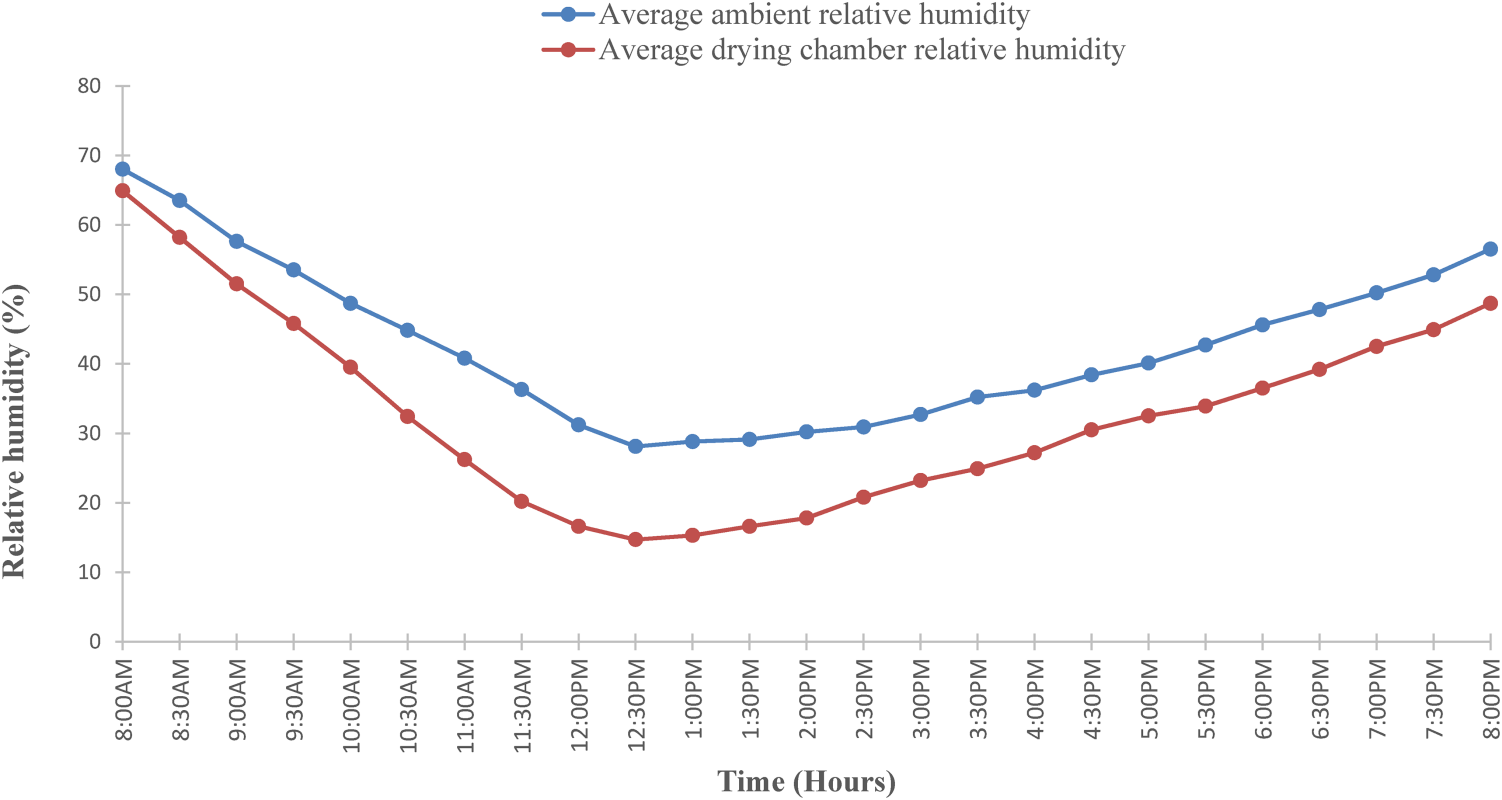
Variation of ambient relative humidity in the drying chamber.

The humidity in the drying chamber and the ambient relative humidity are shown in Figure 6. The ambient relative humidity varies from 25.2 to 65.5%, whereas the relative humidity inside the drying chamber varies from 15.3 to 65%. The relative humidity in the drying chamber was lower than ambient humidity because the evaporator acts as a dehumidifier, reducing humidity in the drying chamber. In this particular study, it was observed that relative humidity inside the drying chamber was significantly low during a day when solar radiation was at its maximum and gradually increased after sunset. This study's findings are in agreement with those of Yahya (2016), who used a solar‐assisted heat pump integrated with a biomass furnace for chilli drying. In that particular study, it was reported that the humidity in the drying chamber was lower compared with the ambient humidity, with a difference of about 47.3%. Therefore, evaporators and solar collectors play an important role in controlling relative humidity during drying.

#### 3.1.1 Drying kinetics

**FIGURE 7:**
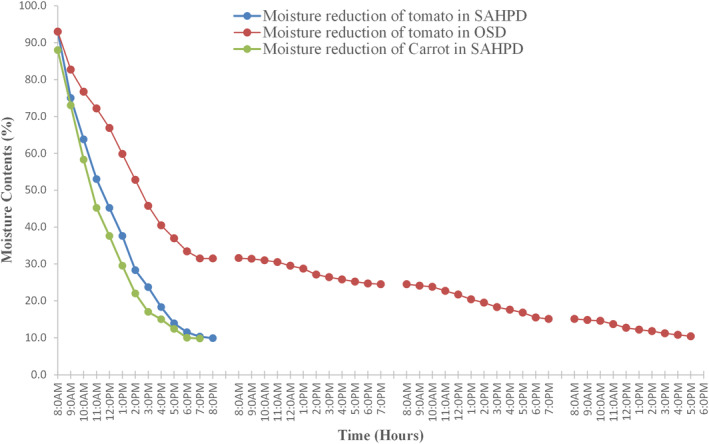
Moisture reduction of tomato and carrot in the solar‐assisted heat pump dryer (SAHPD) and open‐sun drying (OSD).

The percentage of moisture reduction for carrots and tomatoes in SAHPD and OSD is shown in Figure 7. The initial moisture content for tomatoes and carrots decreased from 93% and 88% to 10% in 12 and 11 h, respectively. The drying rate in the SAHPD dryer was higher at the initial stage and continuously declined with time; a similar situation happened on the OSD, in which the rate of moisture reduction was higher on the first day and relatively slow in the following days. The higher drying rate at the initial stage is caused by the loss of water from the surface of the slices of the drying products, which enables quick water flow and evaporation. The initial moisture contents of the second and third days of the tomatoes dried in the open sun were slightly higher as compared to the final moisture contents obtained on the second and third days because of the hygroscopic behavior of tomatoes, in which they tend to absorb water during the night when humidity increases. The hygroscopic behavior was also reported by Das Purkayastha et al. (2013) for drying tomato slices and Owureku‐Asare et al. (2022) during the quality assessment of solar‐dried tomato slices. It was reported that dried products increased in water contents during the following drying days, which was caused by the absorption of water from high‐humidity air.

**FIGURE 8:**
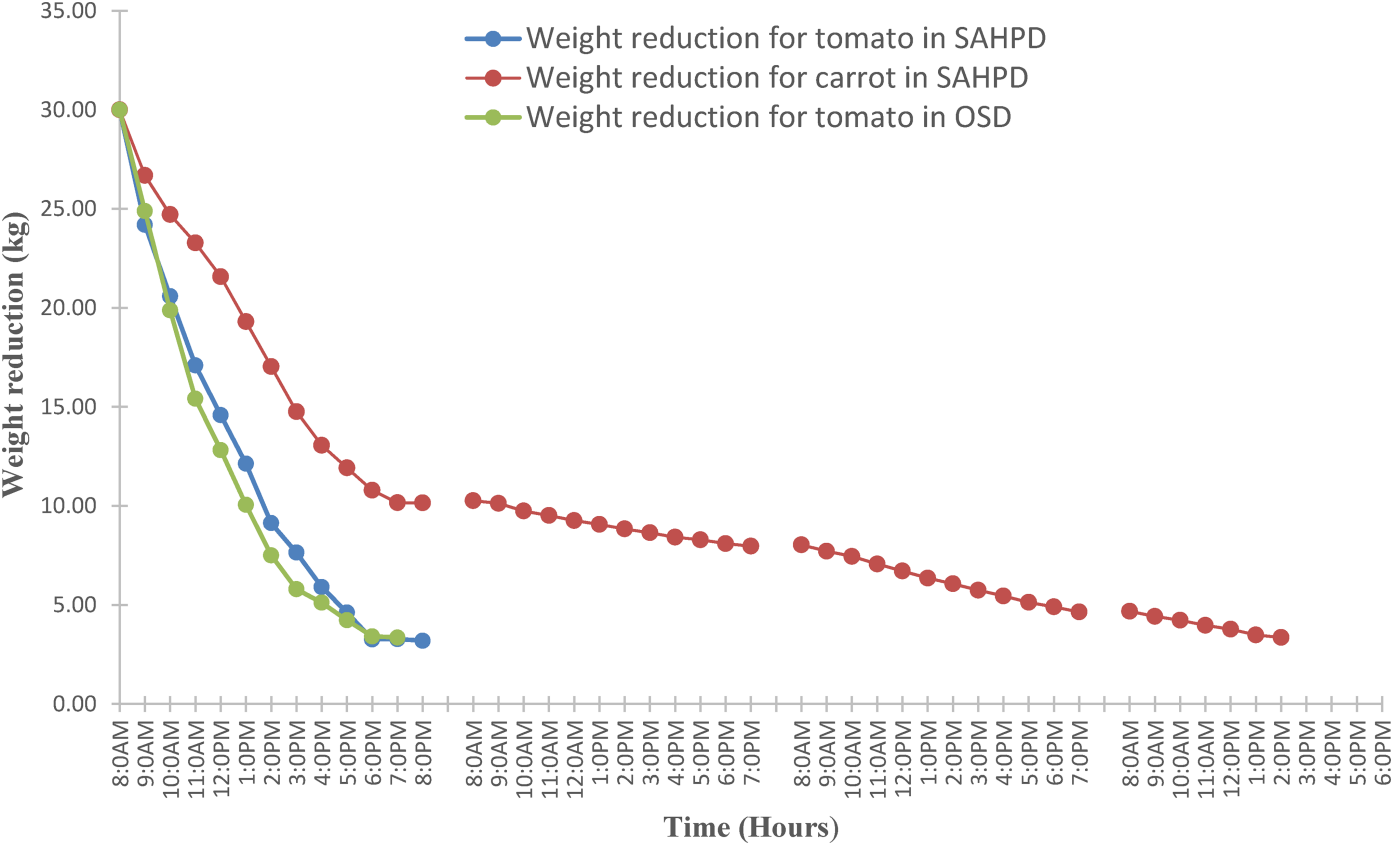
Weight reduction of tomato and carrot in the solar‐assisted heat pump dryer (SAHPD) and open‐sun drying (OSD).

Figure 8 shows the variation of weight reduction with time for tomatoes and carrots using SAHPD and OSD. The fresh sample of 30 kg was dried in the SAHPD at different times, and the final weight of the dried products was 3.3 and 4 kg for tomatoes and carrots, respectively. The time taken to reduce the fresh weight to the final weight was 12 h for tomatoes and 11 h for carrots. The drying time using SAHPD is shorter compared to the 42 h used in OSD to dry tomatoes and carrots. The drying time of 12 h for tomatoes in this study is in agreement with the results obtained by Coşkun et al. (2017), who dried tomato slices using a closed‐loop heat pump dryer in Table 4A. In that particular study, the tomato slices were dried at three different temperatures (35, 40, and 45°C), and the time taken to dry tomatoes to a final moisture content less than 10% was 12, 10, and 7 h, respectively. However, the drying time of 12 h for tomatoes from this work differs from the results obtained by Karabacak and Atalay (2010) in Table 4A. It was reported that tomatoes dried in 78 h in HPD and 148 h in OSD. This is because the tomatoes were sliced quarterly, whereas in this particular study, the tomatoes were sliced into 4‐mm thickness. According to Sadin et al. (2014), drying rate increases with a reduction in product thickness. In comparison with carrots drying. The results in this study are slightly different from those of Aktaş et al. (2017) in Table 4A, who used HPD and an infrared‐heat pump dryer for carrot drying. It was reported that the moisture content of carrots was reduced from 7.06 g water/g dry to 0.14 g water/dry matter in 6.8 h at 40°C and 5.8 h at 50°C. The difference could be due to less quantity of the annually dried products in the dryer, in which it was reported 45.99 kg compared to 1,200 kg of dried carrots, hence making less annual savings.

**TABLE 4A:** Selected studies on the performance of solar‐assisted heat pump dryer (SAHPD) and open‐sun drying (OSD) for drying tomatoes and carrots.

S/N	Dyer type	Product dried	Load (kg)	Drying time (h)		Drying temp (°C)		SMER (kg/kWh)	COP	Thermal efficiency (*η*)	References
				Dryer	OSD	Dryer	OSD				
1	HPD	Tomatoes	20	78	148	–	–	1.573	–	–	Karabacak and Atalay (2010)
2	Vacuum assisted solar dryer	Tomatoes	5	6	7.5	48	30	–	–	–	Rajkumar et al. (2007)
3	HPD	Carrots	–	6.8 and 5.8		45 and 50	–	–	2.96	–	Aktaş et al. (2017)
4	HPD	Tomatoes	2.1	12,10,7	–	35,40,45	–	0.324	2.71	–	Coşkun et al. (2017)
5	SAHPD	Tomatoes and carrots	30	12 and 11	42	45–65	20–30	0.82	3.4	54.0	Current work

The weight of water evaporated during the drying of 30 kg of tomatoes and carrots was calculated using Equation (5) and found to be 26.7 kg for tomatoes and 26 kg for carrots. The drying rate was calculated from Equation (6) and found to be 2.2 and 2.3 kg/h for tomatoes and carrots, respectively. The SMER was calculated from Equation 7 and found to be 1.29 kg/kWh for tomatoes and 1.33 kg/kWh for carrots. The SMER of 1.33 kg/kWh in this study is within the range of 0.156–9.25 kg/kWh reported by Loemba et al. (2022) for most of the heat pump dryers. The COP was calculated from Equation 8 and found to be 3.4, and the thermal efficiency was calculated from Equation 9 and found to be 54.0%. The COP obtained from this study is within the range reported by Loemba et al. (2022) during the compressive assessment of heat pumps for agricultural products, in which it was reported that most of the heat pumps have COPs ranging from 1.94 to 5.38. The thermal efficiency of SAHPD obtained in this study is in agreement with the 56.69% reported by Singh et al. (2020b) in Table 4B during the application of SAHPD for the drying of banana chips.

**TABLE 4B:** Selected studies on the performance of solar‐assisted heat pump dryer (SAHPD) and open‐sun drying (OSD) for drying other vegetables and fruits.

S/N	Dyer type	Product dried	Load (kg)	Drying time (h)		Drying temp (°C)		SMER (kg/kWh)	COP	Thermal efficiency (η)	References
				Dryer	OSD	Dryer	OSD				
1	SAHPD	Mangoes	80	16.3	–	–	–	2.05	3.69	33.4	Wang et al. (2019)
2	SAHPD	Red Chili	22	11	62	70.5		0.14	3.84	9.03	Yahya (2016)
3	SAHPD	Rice	11	0.3	–	61.4–80.9	–	3	3.7	15.4	Yahya et al. (2018)
4	SAHPD	Banana chips	13.5	4	–	48.15	–	1.417	3.43	56.69	Singh et al. (2020b)
5	SAHPD	Chili peppers	10	24		53		2.37	3.17	31.2	Naemsai et al. (2019)
6	SAHPD	Grain	3760	42	–	26.4–41.8	–	1.934	5.03	40.27	Gu et al. (2022)
7	SAHPD	Curcuma	30.7	8	–	57.7	–	2.07	4.35	34.3	Hasibuan et al. (2020)
8	SAHPD	Pineapples	0.6	5	–	37, 40, 43	–	0.218, 0.23, 0.26	2.9, 3.1 and 3.25	–	Salehi (2021)
9	HPD	Mushroom	90	11	–	55	–	2.3	–	–	Juan et al. (2013)
10	SAHPD	Cassava	30.8	9	–	45	–	0.38	3.38	30.9	Yahya et al. (2016)
11	SAHPD	Banana		21		48–52	–	0.6	2.72	–	Kuan et al. (2019)

Table 4A shows some selected studies on the performance of HPD and drying on OSD for tomatoes and carrots. It can be seen from the table that most of the studies have focused on the application of heat pumps alone to the drying of carrots or tomatoes and not a combination of heat pumps and solar energy. Table 4B shows a few selected studies on the performance of SAHPD and OSD for drying other vegetables and fruits using SAHPD. It can be seen from the table that most of the studies have concentrated on the performance of SAHPD using other agricultural products, particularly bananas, and not tomatoes and carrots. From the table, the SMER of the selected studies ranges for SAHPD ranges from 0.6 to 3 kg/kWh, and the COP ranges from 2.72 to 5.03 and thermal efficiency from 9.03 to 56.99. These results are in agreements with the results of the current research under study.

### 3.2 Economic analysis of the SAHPD

The entire initial capital investment, which includes the cost of materials for SAHPD fabrication, was first established, and the total cost was approximately $5222.8, as shown in Table 5. The capital cost was used to calculate the annualized cost and other subsequent economic parameters.

**TABLE 5:** Capital investment of solar‐assisted heat pump dryer (SAHPD).

S/N	Item description	Unit	Quantity	Unit cost ($)	Amount ($)
1	Procurement of heat pump (compressor, condenser and evaporator)	pcs	1	3913.0	3913.0
2	Solar collector glass 6 mm thickness	pcs	1	130.4	130.4
3	Mild steel sheet (2440 × 1220 × 1.5 mm)	pcs	10	56.5	565.2
4	Rubber gasket	roller	1	43.5	43.5
5	Angle iron (30 × 30 × 3 mm)	pcs	6	13.0	78.0
6	Bolts and nuts	kg	3	4.3	13.0
7	Black paint	Liters	4	1.3	5.2
8	Red oxide primer	Liters	10	1.3	13.0
9	Silver paint	Liters	20	1.3	26.1
10	Electrical and electronic control panel	pcs	1	434.8	434.8
	Total cost ($)				5221.8

#### 3.2.1 Annualized cost method

According to Bishoge et al. (2018), Tanzania has potential global horizontal radiation of 4–7 kWh/m^2^/day and sunshine hours ranging from 2800 to 3500 h. However, SAHPD can work even when the sun radiation is not available. For this particular study, the dryer operating time was assumed to be 300 days a year, and the rest of the days were for maintenance of the dryer. Thirty kilograms of fresh tomatoes and thirty kilograms of fresh carrots were loaded in the dryer in consecutive days, and the weight of dried product removed from the dryer per batch was 2.4 and 4 kg for tomatoes and carrots, respectively. The annual weight of dried product was calculated according to Equation (21) and found to be 720 kg for tomatoes and 1200 kg for carrots. The annual capital cost was calculated from Equation (11) and was found to be $569.2. The annualized cost was calculated using Equation (10) and found to be $1076.5. The cost for drying 1 kg of tomatoes and carrots was calculated using Equation (16) and found to be $1.5 and $0.9 for tomato and carrot, respectively.

#### 3.2.2 The life‐cycle savings

This method calculates the daily savings of the SAHPD and then projects the present worth of annual savings for a pre‐determined dyer lifespan. The cost of fresh product per kilogram of dried product was calculated using Equation (18) and found to be $1.8/kg for tomatoes and $1.5/kg for carrots. The total cost of drying 1 kg of the product was computed using Equation (19) and found to be $3.4/kg for tomatoes and $2.4/kg for carrots, respectively. Daily savings (batch‐wise savings) for drying were calculated using Equation (22) and found to be $9.6 tomatoes and $12 for carrots.

The summary of annual savings, Net present worth, and cumulative present worth for tomatoes and carrots for 15 years is depicted in Table 6. The annual savings are $3775 for tomatoes and $4364.9 for carrots. The cumulative present worth is $23,828.8 for tomatoes and $27,553.11 for carrots. Therefore, with the initial investment of $5221.8 for SAHPD, savings of $23,828.8 and $27,553.11 can be obtained over a period of 15 years for tomatoes and carrots, respectively. This result is in agreement with the results reported by Qiu et al. (2016) for drying radish, pepper, and mushrooms using heat recovery and thermal storage in a SAHPD for a lifespan of 20 years. In that particular study, the cumulative net present value for radish, pepper, and mushroom was $10,182, $16,072, and $29,749, respectively. According to Cui et al. (2022), a higher present cumulative value indicates a better project.

**TABLE 6:** Summary of annul savings, NPW, and cumulative for drying tomatoes and carrots.

Tomatoes				Carrots		
Year	Annual savings ($)	Net present worth ($)	Cumulative present worth ($)	Annual savings ($)	Net present worth ($)	Cumulative present worth ($)
1	1957.7	1829.6	1829.6	2263.7	2115.6	2115.6
2	2051.7	1792.1	3621.7	2372.4	2072.2	4187.81
3	2150.3	1755.3	5377.0	2486.4	2029.6	6217.42
4	2253.5	1719.2	7096.2	2605.8	1987.9	8205.34
5	2361.7	1683.9	8780.0	2730.9	1947.1	10,152.41
6	2475.2	1649.3	10,429.3	2862.0	1907.1	12,059.49
7	2594.0	1615.4	12,044.8	2999.4	1867.9	13,927.39
8	2718.6	1582.2	13,627.0	3143.5	1829.5	15,756.90
9	2849.1	1549.7	15,176.7	3294.4	1791.9	17,548.83
10	2985.9	1517.9	16,694.6	3452.6	1755.1	19,303.94
11	3129.3	1486.7	18,181.3	3618.3	1719.0	21,022.99
12	3279.5	1456.2	19,637.4	3792.1	1683.7	22,706.71
13	3437.0	1426.2	21,063.7	3974.1	1649.1	24,355.83
14	3602.0	1396.9	22,460.6	4164.9	1615.2	25,971.07
15	3775.0	1368.2	23,828.8	4364.9	1582.0	27,553.11

#### 3.2.3 Payback period

The payback period was calculated from Equation (26) and found to be 3 years and 2.6 years for tomatoes and carrots, respectively. This means it takes 3 years to recover the initial investment of $5221.8 from the SAHPD for tomatoes and 2.6 years for carrots. The difference between the two is caused by the higher amount of moisture content in the tomatoes (93%), as compared to the carrots (88%). When tomatoes are dried, they remain with less dried matter 2.4 kg whereas carrots remain with 4 kg of dried matter. In this case, carrots have slightly higher annual savings than tomatoes. Hence, drying carrots by using SAHPD could be more profitable as compared to tomatoes. The obtained payback periods are in the range of 1.6–3.6 years for most of the SAHPD, as reported by Loemba et al. (2022) when conducting a comprehensive assessment of heat pump dryers for agricultural products. The payback period in this study is less as compared to the 5 years reported by Xie et al. (2021) for SAHPD with waste heat recovery. The difference could be due to less quantity of the annually dried products in the dryer, in which it was reported 45.99 kg compared to 1,200 kg of dried carrots, hence making less annual savings. Since the dryer is designed for drying vegetables and fruits, the results can also be compared by Qiu et al. (2016) for drying radishes, peppers, and mushrooms using a heat recovery and thermal SAHPD. In that particular study, 10 kg of each radish, pepper, and mushroom were collected by using a heat recovery and thermal storage SAHPD, and the payback periods for radish, pepper, and mushroom were 6 years, 4 years, and 2 years. Since the payback obtained in this study for both tomatoes and carrots is less compared to the investment period of 15 years of the solar dyer's lifespan, this project is attractive for investment.

Table 7 shows a summary of selected studies on the economic analysis of different SAHP drying technologies; however, the annualized cost method is not used in the study because there are limited studies using this method for SAHPD. It can be noted that most studies on economic analysis have concentrated on the assessment of payback periods, with very few focusing on other economic parameters such as annualized costs and life cycle savings.

**TABLE 7:** Some of the previous studies on the economic analysis of different SAHP drying technologies.

S/n	Type of solar dyer	Type of dried product	Weight of the product (kg)	Initial investment cost ($)	Life span	Method used	Findings	Reference
1	SAHPD	Banana chips	13.5	928.52	–	Total annual gain, Return period of investment and payback period	Payback period 3 years. Yearly profit was $95	Singh et al. (2020b)
2	SAHPD with waste Heat Recovery	Carrots	0.731	–	20	Life circle method and payback period	Cumulative present worth of $1988 and payback period of 5 years	Xie et al. (2021)
3	SAHP fluidized bed dryer	Rice	10	2550	10	Net present value an Payback period	Net present value was $8563 an Payback period 1.6	Yahya et al. (2018)
4	Heat recovery and thermal storage solar‐assisted heat pump dryer	Radishes	10	3423	20	Life cycle method and payback period	Cumulative net present value for radish was $10,182, for pepper was $16,072 and mushroom was, $29,749. Payback for radish was 6 years, Pepper 4 years and mushroom 2 years	Qiu et al. (2016)
		Peppers	10					
		Mushroom	10					
5	SAHPD	Tomatoes and carrots	30	5222.6	15	Annualized cost Life cycle savings and Payback period	Annualized cost found $1076.5. Cumulative present worth $23,828.8 for tomatoes and $27,553.11 for carrots. Payback period 3 years for tomatoes and 2.6 years for carrots	This study

## 4 CONCLUSION

A prototype of SAHPD was designed, fabricated, and tested for drying agricultural products. A techno‐economic analysis was conducted for drying tomatoes and carrots. The performance analysis of the SAHPD was analyzed using COP, DT, SMER, and thermal efficiency, and the results were found to be 3.4, 2.3 kg/h, 1.33 kg/kWh, and 54%, respectively. The time taken to dry carrots and tomatoes to their final moisture content was 11 and 12 h, respectively, whereas the time taken to dry tomatoes in OSD was 42 h. The performance analysis revealed that the use of SAHPD in the drying of vegetables and fruits reduces drying time and energy consumption. The economic analysis was analyzed by using the annualized cost, lifecycle savings, and payback period for a life span of 15 years, which was considered the maximum time of dryer operation before disposal. The initial investment in the SAHPD was $5221.8. The annualized cost was found to be $1076.5. The cumulative present worth for 15 years was found to be $23,828.8 and $27,553.1 for tomatoes and carrots, respectively. This is a good savings based on the initial investment in the dryer. The payback periods for tomatoes (3 years) and carrots (2.6 years) were in good agreement with previous works. The use of SAHPD in the drying of carrots produces more return as compared to tomatoes. This study has therefore revealed that the SAHPD is economically feasible and is recommended for further investments in order to reduce postharvest loss, particularly in vegetables and fruits, in developing countries. More research is needed, however, to investigate the use of SAHPD for the drying of high‐volume commercial crops such as tobacco and tea. These crops have traditionally consumed large amounts of biomass materials, such as firewood, for curing, which leads to deforestation.

## AUTHOR CONTRIBUTIONS

**Evordius Laurent Rulazi:** Conceptualization (equal); data curation (lead); formal analysis (lead); funding acquisition (supporting); investigation (equal); methodology (equal); project administration (supporting); resources (supporting); software (equal); supervision (supporting); validation (lead); visualization (lead); writing – original draft (lead); writing – review and editing (supporting). **Baraka Kichonge:** Conceptualization (equal); data curation (supporting); formal analysis (supporting); funding acquisition (supporting); investigation (equal); methodology (equal); project administration (equal); resources (supporting); software (equal); supervision (equal); validation (equal); visualization (equal); writing – original draft (supporting); writing – review and editing (supporting). **Janeth Marwa:** Conceptualization (supporting); data curation (supporting); formal analysis (supporting); funding acquisition (supporting); investigation (supporting); methodology (equal); resources (equal); software (equal); supervision (equal); validation (equal); visualization (supporting); writing – original draft (supporting); writing – review and editing (supporting). **Thomas T. Kivevele:** Conceptualization (lead); data curation (equal); formal analysis (equal); funding acquisition (lead); investigation (equal); methodology (equal); project administration (lead); resources (lead); software (equal); supervision (lead); validation (equal); visualization (equal); writing – original draft (supporting); writing – review and editing (supporting).

## CONFLICT OF INTEREST STATEMENT

The authors declare no conflict of interest.

## Data Availability

The data that support the findings of this research study are available upon request.
